# Performance assessment of carbon based on lignocellulosic material as an effective biosorbent for elimination of iron and manganese from monazite leachate

**DOI:** 10.1038/s41598-026-52221-3

**Published:** 2026-07-02

**Authors:** G. E. Sharaf El-Deen, B. S. Girgis, S. E. A. Sharaf El-Deen

**Affiliations:** 1 Department of Radioactive Waste Treatment, Hot Laboratories Center, Egyptian Atomic Energy Authority, Cairo, 13759 Egypt; 2 Surface Chemistry and Catalysis Lab, National Research Center, El Buhouth Street, P.O. Box 12311, Dokki, Cairo, Egypt; 3 Department of Nuclear Chemistry, Hot Laboratories Center, Egyptian Atomic Energy Authority, Cairo, 13759 Egypt

**Keywords:** Sorption and separation, Rare earth elements, Fe^3+^ and Mn^2+^, Carbon based on lignocellulosic cotton stalks, Monazite leachate, Chemistry, Environmental sciences, Materials science

## Abstract

The purification of REEs from leaching liquor of monazite ore; which contains contaminated iron and manganese, are significant important for REEs applications. Herein, this work investigated the sorption and separation of Fe^3+^ and Mn^2+^ from both aquatic solution and REEs of monazite liquor using carbon-based on lignocellulosic cotton stalk material (CCS) as a green biosorbent at optimum conditions. The prepared CCS was characterized by different physicochemical and morphological techniques as FTIR, SEM, TEM, and specific surface area measurement, which was 831 m^2^ g^− 1^ with total pore volume; 0.431 m^3^ g^− 1^, micro-pores percentage; 93.27%, and meso-pores percentage; 6.73%. The sorption investigations like pH, stirring time, initial metal-ions concentration, temperature, and adsorbent weight were carried out to identify the best reaction parameters. The obtained results were analyzed using different kinetic and isotherm patterns. The pseudo-second order kinetic model surpasses on the other kinetic models in the sorption process, indicating the chemisorption reaction. The sorption isotherm matched with Langmuir model, depending on the highest R^2^ and lowest of error functions. The maximum monolayer sorption capacities of Fe^3+^ and Mn^2+^ ions onto CCS are 531.9 and 680.27 mg/g, respectively at optimum sorption conditions (pH 2.6 for Fe^3+^ and pH 5.5 for Mn^2+^, stirring time 30 min. at 25 °C). The sorption approach was exothermic, randomness and spontaneous in nature. Lastly, CCS can be highly separated Fe^3+^ and Mn^2+^ from REEs of monazite leachate with separation factors reached 228.947 and 2.5735, respectively. Consequently, the low-cost CCS sorbent was utilized for purification of REEs from Fe^3+^ and Mn^2+^ pollutants of monazite ore liquor.

## Introduction

Rare earth elements play an essential role in modern industries, leading to increased interest in the sources containing these REEs. Rare earth elements are used in many high-tech purposes such as, medical field, electronics, metallurgy, magnets, sensors, communication devices, and nuclear applications^1–4^. It is often found in monazite ores with other heavy metals, this refers to decline and restrict the sorption efficiency of both REEs and metal ions^5^. The pollution with toxic heavy metals is regarded as one of the more significant ecological issues and that greatly affects human health. Therefore, the recovery/separation of poisonous metal ions like Fe^3+^ and Mn^2+^ from rare earth element solutions is of utmost importance. Several methods are utilized for elimination and extraction of metal ions and REEs from aqueous solutions to solve this issue, like ion exchange^2,6,7^, membrane^8^, co-precipitation^3,9^, hydrogen reduction^10^, solvent extraction^6,7,11–13^ and adsorption^14^. The adsorption method is vastly utilized, because of its low-cost, simple process with high efficiency. Due to the large availability of natural materials, and some agricultural waste, many researchers around the world have become interested in using them to remove heavy metal ions from polluted water. This is due to their low cost, widespread availability, renewable and unused resources, and environmental friendliness, as well as their unique properties. Among these natural materials are cotton stalks. However, burning cotton stalks in the traditional way leads to serious environmental problems. Therefore, researchers have focused on converting cotton stalks into activated carbon material that can be used to purify polluted water from heavy metals, dyes, and other contaminants. Girgis et al. (2009)^15^ prepared activated carbon from cotton stalks (BR1) using 60% H_3_PO_4_, (impregnation ratio 0.1, soaking time 22 h) at 420 °C, achieving sorption capacities of 21.7, 824, and 104 mg/g for Pb(II), iodine, and MB, respectively. The second sorbent (BR2), synthesized with a 0.2 impregnation ratio and 44 h soaking time, clarified capacities of 565 mg/g for iodine and 180 mg/g for MB. Mahmoud et al. (2014)^16^ evaluated the behavior of AC and AC-NaLS that prepared by 50% H_3_PO_4_ at 420 °C for the sorption of Ce(IV), exhibited adsorption capacities of 0.0347 and 0.0576 mmol/g, respectively. The activated carbons derived from different precursors (CS 55, DP 55, PS 55, ALS 55, and OS 55) were manufactured by Daifullah et al. (2003)^17^ using 50% H_3_PO_4_ and heat treatment at 773 K and utilized to adsorb BTEX compounds with removal capacities reached 27.7, 25.6, 26.5, 20.2, and 27.9 mg/g, respectively. In 2011, El Sherif et al.^18^ used an activating agent (50% H_3_PO_4_) at 500 °C to prepare a CSP material for adsorbing Cd^2+^ (31.6 mg/g) and Ni^2+^ (33.5 mg/g). While chemical activation using alkaline agents was employed by Fathy et al. (2010)^19^, that synthesized various ACs (CCS-1K700, CCS-1K750, CCS-1K800, and CCS-4K800) under activation by KOH at different temperatures (700 °C, 750 °C, and 800 °C), illustrating that the maximum iodine adsorption capacities were 594, 663, 743, and 426 mg/g, respectively and for MB, reached to 198 mg/g onto CCS-1K750 and 222 mg/g onto CCS-1K800. Also, Deng et al. (2010)^20^ prepared two activated carbons (KAC and KCAC) from cotton stalk by microwave assisted KOH and K_2_CO_3_ activation for removal of MB, achieving removal capacities of 294.12 and 285.71 mg/g, respectively.

Furthermore, various adsorbent materials have been used for the recovery of REEs and heavy metals. For instance, according to the natural adsorbent materials; The adsorption of iron and manganese from acid mine drainage by zalacca (Salacca zalacca) peel- activated carbon, carbonized at 300 °C for 30 min. and activated with KOH has been studied by Anifah E. M. et al. (2024)^21^. It was illustrated that the synthesized activated carbon adsorbed 80% and 24% for iron and manganese, respectively at shaking time, 60 min., and adsorbent weight, 0.8 g/100 ml. Brishti R. S. et al. (2023)^22^, employed the activated carbon that prepared from Bombax ceiba fruit shell with chemical activation by zinc chloride for removal of Fe^3+^ from aqutic media. The adsorption capacity of Fe^3+^ using the manufactured activated carbon reached 37.16 mg g^− 1^. Tarigan E.R. et al. (2025)^23^, investigated the adsorption mechanism of heavy metals (Cu, Pb, Fe and Zn) using activated carbon derived from Hydrilla verticillata. Where, the adsorption capacity reached to 6.8, 9, 15.9, and 18 mg g^− 1^ for Zn, Cu, Fe, and Pb, respectively. Beleño Cabarcas, M.T. et al. (2024)^24^, used cotton stalk as an adsorbent for sorption of Copper(II) Ions from wastewater. The removal % of Cu^2+^ was 66.5% at initial concentration 50 mg L^− 1^, pH 5.5, adsorbent weight 0.6 g, and shaking time 60 min. The uptake % of wastewater samples that contains on both agricultural and livestock was 87.60% and 85.05%, consecutively, at initial Cu concentration of 25 mg/L.

While, the adsorbents that utilized for recovery of REEs like, Municipal solid waste incineration ash (MSWIA), Citrate, oxalate, and zeolite that created from citrate residue and oxalate filtrate were utilized by Wen Y. et al. (2024)^25^ as a possible substitute for REEs recovery. The results indicated that, Citrate extracted > 80% of the total REEs at pH 2.0. While, oxalate can concentrate more than 98% of extracted REEs by ∼7 − 12 times compared to raw MSWIA. In addition to the prepared zeolites reduced the total solid waste volume to about 80% with immobilization efficiency of heavy metals reached to 75%, indicating the recovery of REEs. Shahr El-Din et al. (2021)^26^ utilized a calcium/alginate-graphene oxide (Ca/Alg–GO) gel beads to eliminate thorium, iron, and uranium from the high grade monazite concentrates. The results indicated that the highest adsorption ability of U^6+^, Fe^3+^, and Th^4+^ were 129.0 mg g^− 1^, 139.0 mg g^− 1^, and 418.0 mg g^− 1^, consecutively on Ca/Alg–GO gel beads. Hassan MR et al. (2024)^27^, explained the recovery of Ce^+ 3^ and Fe^+ 3^ from nitric acid aqueous media using manganese- substituted- cobalt- ferrite nanoparticles (MCFO-NPs) sorbent and the maximum adsorption capacities of Ce^+ 3^ and Fe^+ 3^ were 130.0 and 161.0 mg/g, respectively. Abu Elgoud et al. (2022)^5^ utilized the composite of graphene oxide-citrate (GO-C) for removal of Mn^2+^, Fe^3+^, and Ni^2+^ from Lanthanide aquatic media. The results indicated altitude sorption capability for manganese, iron, and lead onto GO-C composite in existence of lanthanides. In addition to, the maximum sorption capacities from Langmuir model were 171.23, 216.45, and 531.91 mg/g for Ni^2+^, Mn^2+^, and Fe^3+^, respectively. Abass M.R. et al. (2024)^28^, used antimony oxide (Sb_2_O_5_) sorbent for extraction of Y^3+^, Ce^3+^, and Gd^3+^ from both aquatic medium and high-grade monazite (HGM), Their studies revealed that the maximum capacities of Gd^3+^, Y^3+^, and Ce^3+^ were 24.1, 30.6, 27.5 mg/g, respectively. Furthermore, the sorption isotherm results are very relevant to a Langmuir model with maximum monolayer capacities of 19.8, 23.2, and 27.9 mg/g for Gd(III), Ce(III), and Y(III), respectively.

The aforementioned literature indicates that, although these studies have demonstrated the sorption efficiency of Fe^3+^ and Mn^2+^ from aquatic medium and wastewater using various lignocellulosic sorbent materials, they have not been connected to practical applications. It was also noticed that neither our CCS material nor activated carbons had ever been employed in the separation of these elements from REEs of ores liquors. Therefore, in this work, the authors converted the precursor material of CS to CCS as an effective biosorbent for elimination of Fe^3+^ and Mn^2+^, depending on its large surface area with the presence of many active groups on the adsorbent surface. Then, CCS was characterized using FTIR, SEM and S_BET_ to determine the adsorption mechanism. The novelty of this work lies on the purification/separation of Mn^2+^ and Fe^3+^ ions in-particular from REEs in monazite liquor. Where, the iron is the most prevalent metal contaminants in monazite leachate fluid. This highlights the importance of using the CCS adsorbent in practical applications, which represents a novel application in this field.

## Materials and methods

### Preparation of CCS

**Fig. 1:**
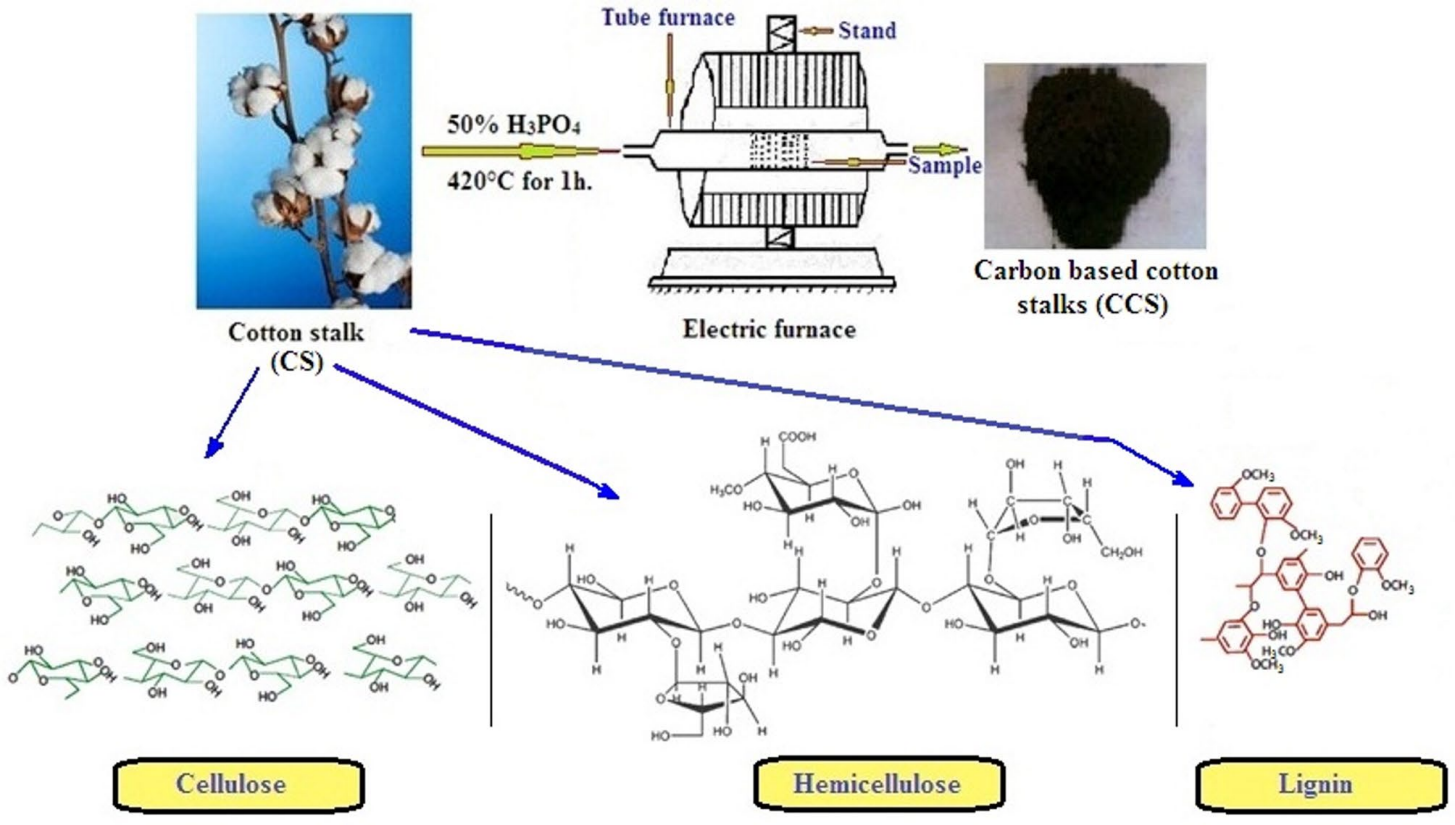
A schematic diagram of the system used for preparation of CCS and the approximate composition of CS.

The local cotton stalk (CS) was selected as a precursor material for synthesis of CCS. Firstly, cotton stalks were air-dried and then cut into small pieces. Secondly, 20 g of CS was soaked in 50% H_3_PO_4_ in a volume enough to cover the CS completely with slightly stirring to ensure that the acid penetrated throughout the CS, and left overnight at room temperature. Thirdly, the impregnated CS was dried at 120 °C for 5 h., then transferred to the stainless steel tube reactor as shown in Fig. 1 and heated up to 420 °C with gradually rate of 50 °C/10min and hold time 1 h. After carbonization, The cooled carbon product was thoroughly washed several times using hot distilled water to eliminate the excess H_3_PO_4_ and open the pores produced from the activation process till the pH of the washing solution become neutral. Eventually, it was dried at 110 °C for 6 h. The final prepared material was named as CCS^15,16,29,30^. H_3_PO_4_ was chosen as an activating agent, which increases the mixed-type of porosity (as, micro- and meso-pores)^29,31,32^. In addition to, during the soaking, H_3_PO_4_ diffuses into CS, and during the carbonization process at 420 °C, part of H_3_PO_4_ is converted to dehydrated form and enters in the micro-pores and deposited in it, which difficult to remove it completely by the sequentially washing. In addition to, it’s probable difficulty of resolubility. Therefore, the phosphate groups are appeared in the CCS^29,31^.

### Techniques of characterization

The porosity of CCS was explored by adsorption-desorption isotherm at 77 K under N_2_-gas with employing the analyzer of surface area (model; Nova 1000e series, USA). The texture factors were illustrated depending on the Brunauer- Emmett-Teller (BET)-relation, total pore volume (V_p_) was clarified from amount of N_2_ hold as liquid at P/P^o^= 0.95, and average pore radius (Rp) from Rp= (2Vp)/ S_BET_.

The qualitative estimation of the O-functionalities and other group was detected by recording the FTIR spectra within 400–4000 cm^-1^ range depending on the equipment of Fourier transform infra-red spectroscopy of model Perkin Elmer Spectrum 100.

The synthesized activated carbon’s surface morphology was verified with a JEOL analytical scanning electron microscope (SEM) (JSM-5400, Japan). A 25 KV accelerating voltage was used for the scan.

### Metal salts and apparatus

Whole the reagents used in this study are high-purity chemicals (AR grade). The solutions of Fe^3+^ and Mn^2+^ were produced from dissolving FeCl_3_.6H_2_O and MnCl_2_. H_2_O (which were obtained from Sigma-Aldrich) in deionized water. NaOH (from ADWIC) and HCl (from Merck) were used to adjust the pH of the solution, and the pH meter of model 710, Orion, USA was employed to measure the pH values. The concentrations of metal ions were detected by atomic absorption spectroscopy (Hitachi Z-8100, Germany).

### Sorption process

The sorption behavior of both Fe^3+^ and Mn^2+^-ions on CCS was studied to improve their separation; particularly Fe^3+^ from rare earth elements of monazite leachate liquor. Sorption studies were performed in batch manner by shaking 10 mg of the synthesized material with 50 ml of metal ions concentrations ranging (10–50 mg/l), pHs (1–9), contact time (2–20 h), and at temperatures (298–328 k). After shaking period, the solution was filtered using Whatman filter papers No.41 and the concentration of metal ions before and after shaking was measured by atomic absorption spectrophotometry. The practical sorption experiments were duplicated twice. The sorption % and the adsorbed amount (q_e_, mg/g) were calculated using Eqs. (1, 2) as follows:

1 $$\:\mathrm{S}\mathrm{o}\mathrm{r}\mathrm{p}\mathrm{t}\mathrm{i}\mathrm{o}\mathrm{n}\:{\%}=\left(\frac{{\mathrm{C}}_{\mathrm{o}}-\:{\mathrm{C}}_{\mathrm{e}}}{{\mathrm{C}}_{\mathrm{o}}}\right)\times 100\:$$

2 $$\:{\mathrm{q}}_{\mathrm{e}}=\frac{\mathrm{V}}{\mathrm{m}}{(\mathrm{C}}_{\mathrm{o}}-\:{\mathrm{C}}_{\mathrm{e}})$$

where C_o_ and C_e_ are the initial and equilibrium concentrations of the metal ion in the solution (mg/L), V is the solution volume (L) and m is the mass of adsorbent (g).

### **Adsorption isotherm and kinetic models**

The mechanism and efficiency of Fe^3+^ and Mn^2+^ sorption onto CCS can be largely determined, through assessing the applicability of kinetic and isotherm adsorption models. An isotherm adsorption model describes how the adsorbent surface and the adsorbate particles interact. While, the kinetic models indicates the mathematical description of the rate of reaction for the adsorption process, depending on the changes in time and temperature parameters of the experimental operation. In addition to, these models could be used to illustrate whether the sorption reaction is a chemical or physical reaction. To realize these targets, four kinetic models like, pseudo-first-order (PFO), pseudo-second-order (PSO), Elovich and intra-particle diffusion as well as isotherm models, containing Langmuir, Freundlich, and Dubinin-Radushkevitch (D-R) models were applied. The linear equations used for the isothermal and kinetic sorption models are listed in Table 1.

### Error equations

To foretell the best model, through its well fit and low error rate among the practical experiments data and the prophesy isotherm, indicates that the CCS reacts with both of Fe^3+^ and Mn^2+^ very well. Various types of error equations utilized to discover the better-fitting model include Standard deviation (SD), Residual sum of squares (RSS), Error mean square (EMS), and Correlation coefficient (R^2^) and are listed in Table 2.

**Table 1:** Kinetics and equilibrium isotherm models were used for Fe^3+^ and Mn^2+^ sorption onto CCS.

Isotherm models	Linear form	Graph	Meaning of the parameters
Langmuir^33^	$\:\frac{1}{{\mathrm{q}}_{\mathrm{e}}}=\frac{1}{{\mathrm{q}}_{\mathrm{m}\mathrm{a}\mathrm{x}}}+\left(\frac{1}{{\mathrm{b}\mathrm{q}}_{\mathrm{m}\mathrm{a}\mathrm{x}}}\right)\left(\frac{1}{{\mathrm{C}}_{\mathrm{e}}}\right)$	1/q_e_ against 1/C_e_	q_e_ is the amount of metal ions adsorbed at equilibrium (mg g^-1^). q_max_ is the maximum adsorption capacity of metal ions (mg g^-1^) to form a monolayer of adsorbate (Fe^3+^ or Mn^2+^) on the homogeneous surface of CCS adsorbent. C_e_ is the concentration of metal ions at equilibrium (mg L^-1^). b is the Langmuir constant that related to the adsorption energy (L.mg^-1^)
Freundlich^34^	q_e_ = k_f_ C_e_^1/n^ $\mathrm{log}{\mathrm{q}}_{\mathrm{e}}=\mathrm{log}{\mathrm{k}}_{\mathrm{f}}+\left(\frac{1}{\mathrm{n}}\:\right)\mathrm{log}{\mathrm{C}}_{\mathrm{e}\:\:\:\:}$	log q_e_ against log C_e_	q_e_ is the amount of metal ions adsorbed at equilibrium (mg g^-1^). C_e_ is the concentration of metal ions at equilibrium (mg L^-1^). K_f_ (mg g^-1^) and n are Freundlich constants that refers to the adsorption capacity and the intensity of adsorption, respectively.
Dubinin- Radushkevich (D-R)^35,36^	$\:\mathrm{l}\mathrm{n}{\mathrm{q}}_{\mathrm{e}}=\mathrm{l}\mathrm{n}{\mathrm{q}}_{\mathrm{m}}-{\upbeta\:}{{\upepsilon\:}}^{2}$ $\:{\upepsilon\:}=\mathrm{R}\mathrm{T}\:\mathrm{l}\mathrm{n}\left(1+\frac{1}{{\mathrm{C}}_{\mathrm{e}}}\right)$ $\:\mathrm{E}=\frac{1}{\sqrt{2\:{\upbeta\:}}}$	lnq_e_ against$\:{{\upepsilon\:}}^{2}$	q_e_ is the adsorption capacity (mol/g). $\:{\mathrm{q}}_{\mathrm{m}}$is the maximum adsorption capacity, i.e. the amount of metal ions at complete monolayer coverage (mol/g). $\:{\upbeta\:}$(mol^2^.kJ^-2^) is constant of D-R that refers to the sorption energy. ε is the Polanyi potential. C_e_ is the concentration of metal ions at equilibrium (mg L^-1^). T (K), R (J.mol^-1^.K^-1^), and E (kJ.mol) are the temperature in Kelvin, the ideal gas constant (8.314) and the sorption energy to transfer one mole of the sorbate from the solution to the solid surface, respectively.
Kinetic models	Linear form	Graph	Meaning of the parameters
Pseudo-First-order (PFO)^37^	$\:\mathrm{log}({\mathrm{q}}_{\mathrm{e}}-{\mathrm{q}}_{\mathrm{t}})=\mathrm{log}{\mathrm{q}}_{\mathrm{e}}-\frac{{\mathrm{K}}_{1}}{2.303}\:\mathrm{t}$	$\:\mathrm{log}({\mathrm{q}}_{\mathrm{e}}-{\mathrm{q}}_{\mathrm{t}}$) against t	q_e_ (mg g^-1^) and q_t_ (mg g^-1^) are the amount of metal ions adsorbed at equilibrium and at time (t), respectively; $\:{\mathrm{K}}_{1}$describe the adsorption rate constant of pseudo-first-order (min^-1^).
Pseudo-Second-order (PSO)^36^	$\:\frac{\mathrm{t}}{{\mathrm{q}}_{\mathrm{t}}}=\frac{1}{{\mathrm{K}}_{2\:\:}{\mathrm{q}}_{\mathrm{e}}^{2}}+\frac{\mathrm{t}}{{\mathrm{q}}_{\mathrm{e}}}$	t/q_t_ against t	q_e_ (mg g^− 1^) and q_t_ (mg g^− 1^) are the amount of metal ions adsorbed at equilibrium and at time (t), respectively. $\:{\mathrm{K}}_{2}$is the rate constant of the pseudo-second-order relation (g mg^− 1^ min^− 1^). This model predicts the chemisorption reaction.
Intra-particle diffusion (IPD)^35^	$\:{\mathrm{q}}_{\mathrm{t}}={\mathrm{C}+\mathrm{k}}_{\mathrm{i}\mathrm{d}\:\:}{\mathrm{t}}^{0.5}$	$\:{\mathrm{q}}_{\mathrm{t}}$against t^0.5^	q_t_ (mg g^-1^) is the amount of metal ions adsorbed at time (t). K_id_ (mg g^-1^.min^-0.5^) is the intra-particle diffusion rate constant. C (mg g^-1^) is the intercept of the plot which refers to the boundary layer thickness
Elovich^38–40^	$\:{\mathrm{q}}_{\mathrm{t}}=\frac{1}{{\upbeta\:}}\mathrm{l}\mathrm{n}\left({\upalpha\:}\:{\upbeta\:}\right)+\frac{1}{{\upbeta\:}}\mathrm{l}\mathrm{n}\left(\mathrm{t}\right)$	$\:{\mathrm{q}}_{\mathrm{t}}$against ln t	q_t_ (mg g^-1^) is the amount of metal ions adsorbed at time (t). α (mg g^-1^ min^-1^) is the Elovich constant that represents the initial adsorption rate. β (g.mg^-1^) is the Elovich constant that related to the extent of surface coverage and activation energy for chemisorption

**Table 2:** Error equations for sorption of Fe^3+^ and Mn^2+^-ions onto CCS.

Error function	Equation
Error mean square (EMS)^37^	$\:EMS=\frac{1}{n}\:\sum\:_{i=1}^{n}{(q}_{exp.}-{q}_{calc.}{)}^{2}$
Residual sum of squares (RSS)^37,40^	$\:RSS=\:\sum\:_{i=1}^{n}{(q}_{exp.}-{q}_{calc.}{)}^{2}$
Standard deviation (SD)^39^	$\:SD=100\:\times \:\sqrt{\frac{\sum\:[\left({q}_{t}^{exp}-\:{q}_{t}^{cal}\right)/\:{q}_{t}^{exp}{]}^{2}}{n-1}\:}$

$\:{q}_{t}^{exp}$ and $\:{q}_{t}^{cal}$ (mg g^− 1^) are the amounts of Mn^2+^ and Fe^3+^ adsorbed experimentally and calculated at time t, respectively, and n the number of experimental data points.

### Determination of the point of zero charge (pHpzc)

The pH at which the ultimate charge of the adsorbent’s surface equals zero is known as the point of zero charge (pzc), and it is calculated using a batch method. Several flasks of 25 mL containing on 0.1 g of CCS with 10 mL of 0.01 M NaCl, and the pH was adjusted from 1.0 to 9.0 using either 0.1 M HCl or 0.1 M NaOH (pHinitial). The mixtures were then agitated for twenty-four hours, and the pH_final_ of the solutions was determined. pHpzc of CCS was found to be 3.8.

## Results and discussion

### Characterization of adsorbent

#### Function groups of the synthesized adsorbent

**Fig. 2:**
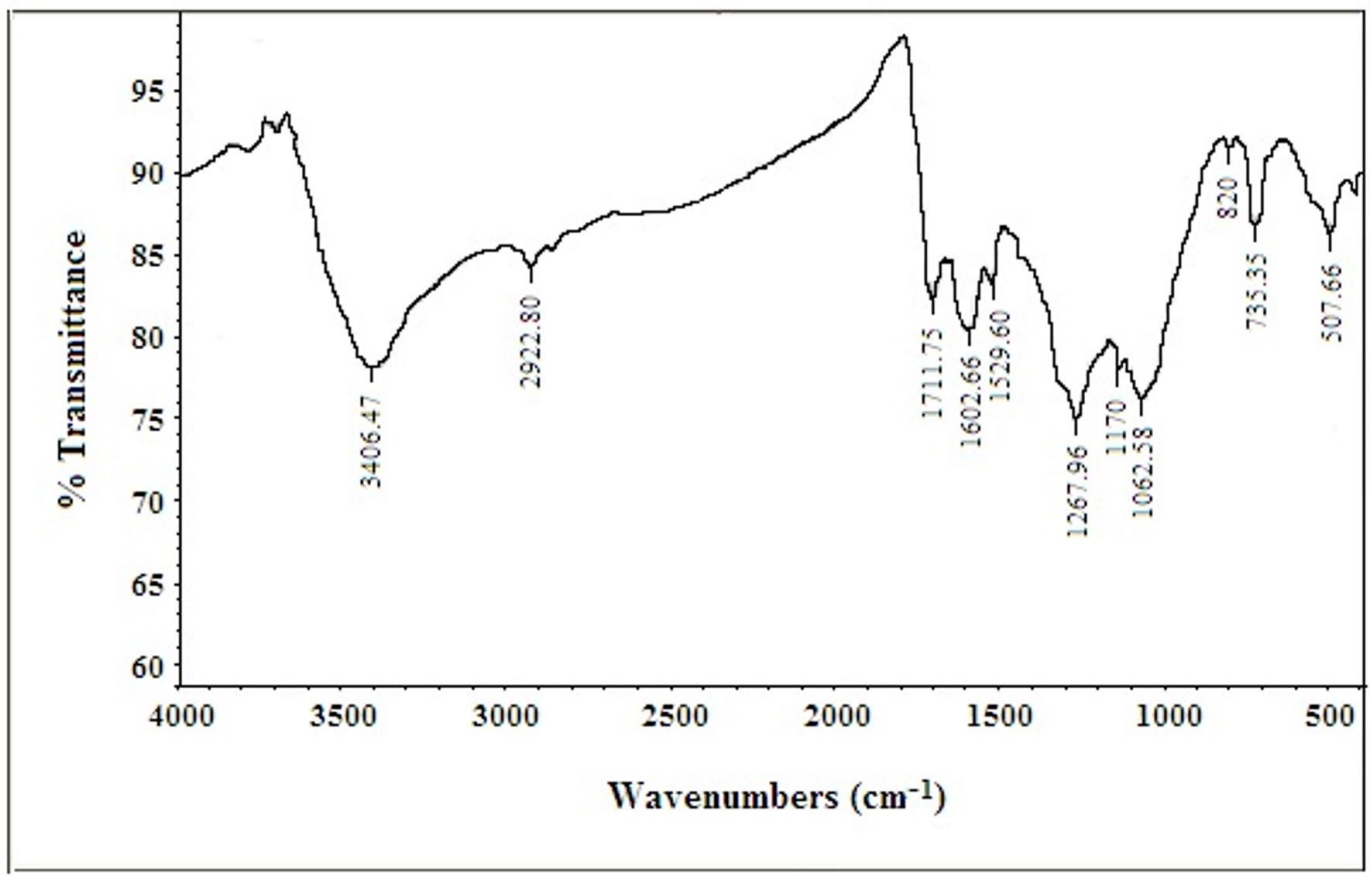
FTIR spectra of CCS.

The distinct surface functional groups on CCS were studied. The main configurations of CS were cellulose, hemicellulose and lignin^41,35^ as shown in Fig. 1. Therefore, the expected oxygen groups in CS are found in the form of hydroxyl, carbonyl, ether, phenol, etc.^42^. According to FTIR spectrum (as shown in Fig. 2, CCS indicated the following bands: the very broad band at 3600–3320 cm^− 1^ with a maximum at about 3406 cm^− 1^ is refers to the O–H stretching vibration of hydroxyl groups from carboxyl, phenols or alcohols that found in cellulose, hemicellulose and lignin, in addition to adsorbed water^43,31^. The small peaks at 2922 and 2870 cm^− 1^ are due to C-H stretching of aliphatic -CH, -CH_2_ and -CH_3_ groups. The bands from 1711 to 1530 cm^− 1^ are assigned to C = O stretching vibrations of ketones, aldehydes, lactones or carboxyl groups and C=C stretching vibration of aromatic ring that appeared at 1530 cm^− 1^^44,45^. The bands at 1300 ~ 1000 cm^− 1^ may be resulted from the activation of the CS raw material by phosphoric acid, forming phosphate groups^44^. The absorption bands within the range 1268 –1170 cm^− 1^ are connected to the existence of P–O–C, stretching vibrations of P = O and P = OOH^44,45^. The band at 1062 cm^− 1^ is related to the symmetrical vibration of P–O–P of polyphosphate and to P^+^-O^−^ ionizing linkage in acid phosphate esters^44,45,32,39^. Bands below ≤ 900 cm^− 1^ are ascribed to out of plane deformation mode of C–H in benzene rings.

#### Scanning (SEM) and transmition electron microscopy (TEM) of CCS

**Fig. 3:**
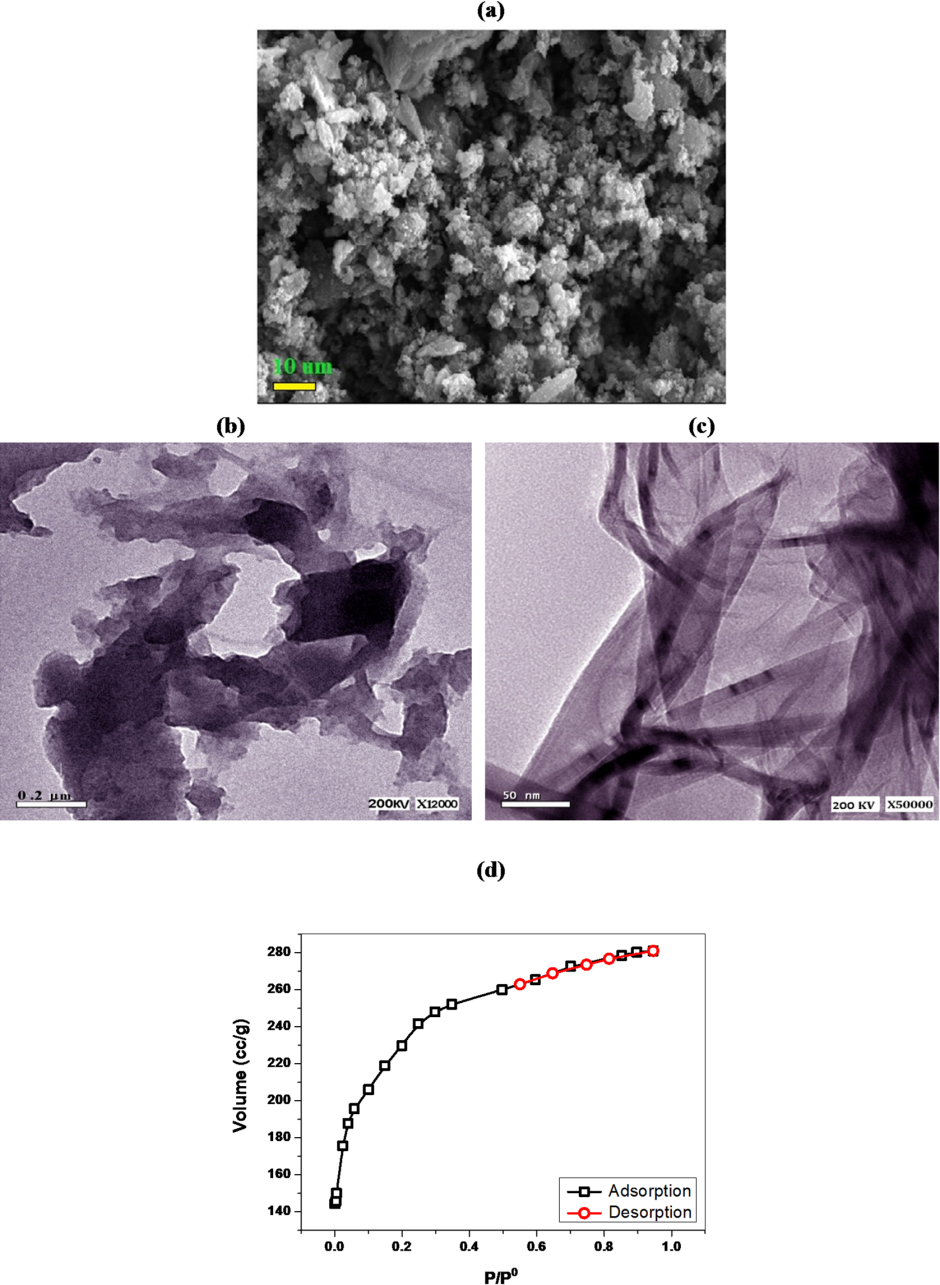
(**a**) SEM, (**b**,**c**) TEM, and (**d**) N_2_/77K adsorption-desorption isotherm of CCS texture.

Scanning Electron Microscope (SEM) of CCS (Fig. 3a) indicated that the CCS appeared as irregular granules with presence a lot of pores on the studied cellulosic material carbon surface. While the morphology of the CCS texture surface was demonstrated by a higher resolution transmition Electron Microscope (TEM) technique as illustrated in Fig. 3b, c. Figure 3b clarified slices shapes of CCS with some large pores between them. Figure 3c provided more explanatory data related to the structure, surface characteristics shown smooth surface texture with narrow rod and thin slices –shapes. These results perhaps attributed to the using of high concentration of H_3_PO_4_ (80%) in the manufacture of CCS texture, which may be suitable for producing many porous on the material surface.

#### Specific surface area and pore structure of CCS adsorbent

Figure 3d displays the N_2_-adsorption-desorption isotherm for CCS adsorbent as a relationship among the proportional pressure (P/P°) and the volume (V), which belonging to type I depending on IUPAC recommendations^46^, indicating that the carbon under investigation is a microporous. Such microporous character is confirmed by the high content of microporosity (93.27%) as indicated in Table 3. The pores of adsorbent can be sorted into micropores (*d* < 2 nm), mesopore (*d* = 2–50 nm) and macropore (*d* > 50 nm), according to the IUPAC classification of pore dimensions^47^. An average pore radius R_p_=10.4 A^o^ (1.04 nm) strongly support that the texture is generally composed of narrow micropores.

**Table 3:** Textural properties of CCS.

Parameter	Value	Parameter	Value
Total surface area (S_t_^α^) (m^2^/g)	831	Mesopore volume (V_meso_) (cm^3^/g)	0.029
Total pore volume (V_p_) (m^3^/g)	0.431	% Mic (V_o_/V_p_)	93.27
Average pore radius (R_p_) (A^o^)	10.40	% Meso (V_mes_./V_p_)	6.73
Micropore volume (V_o_^α^) (cm^3^/g)	0.402		

### Batch sorption studies

#### pH study

The basic parameters that affect the adsorption process are pH, pHpzc and speciation of metal ions in aqueous medium.

**Fig. 4:**
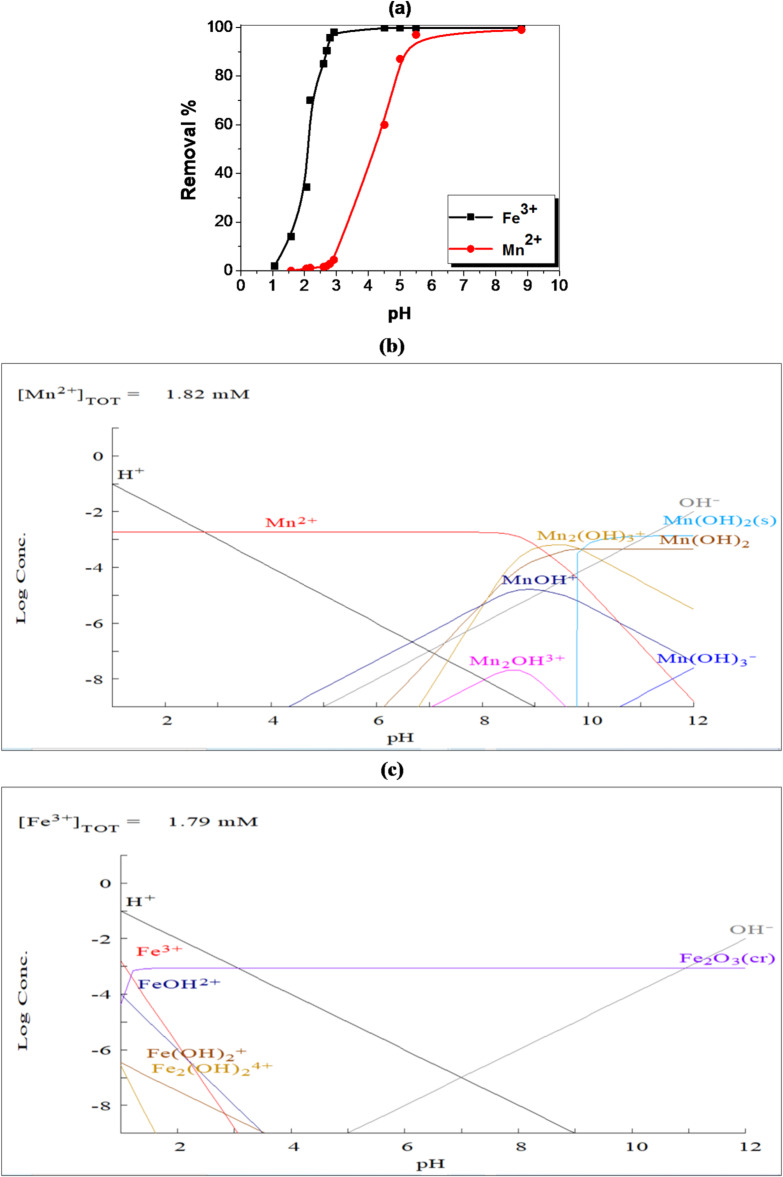
(**a**) Effect of pH on adsorption of Fe^3+^ and Mn^2+^ ions by CCS (C_i_ = 30 mg/L, m = 10 mg, V = 50 ml, T = 25 °C, t = 120 min) and Speciation diagram of (**b**) Mn^2+^ and (**c**) Fe^3+^.

The removal of Fe^3+^ and Mn^2+^ at diverse initial pHs (pH_in_) is illustrated in Fig. 4a. The studied ions’ sorption on CCS increases with increasing pH. According to the adsorption studies, the pH must be less than the pH for precipitation of respective metal ions.

Figure 4a denoted the adsorption of Mn^2+^ at various initial pHs. At low concentrations of manganese(II), it was found to hydrolysis very slightly at pH ≤ 6.2. Whilst, at pH > 6.2, it produces varied species and at pH-values from 9.5 to 10, Mn^2+^ ions precipitate as Mn(OH)_2(s)_ as indicated in Fig. 4b, hence, Mn^2+^ is prevails in the acidic media. Therefore the optimum pH chosen for sorption studies was 5.5.

On the other hand, as explored in Fig. 4a, the adsorption technique of Fe^3+^ rises significantly at pH amongst 1 and 3. But, at pH values greater than 3, both sorption and precipitation occur, as illustrated in Fig. 4c. In acidic media, polyspecies like Fe_2_(OH)_2_^4+^, FeOH^2+^, and Fe(OH)_2_^+^ are created. However, in neutral and basic solutions, Fe(OH)_3_(aq) and Fe(OH)_4_^−^ are founded^48^. Therefore, an ideal pH of 2.6 was selected for the sorption tests.

The pHzpc of CCS is 3.8. This means, the CCS surface has positive charge at pH lower than pHpzc and vice versa. So, the high sorption of Mn^2+^ at pH 5.5 is due to an electrostatic attraction between Mn^2+^ and (-Ve) charge of CCS surface at pH> pHpzc. But for Fe^3+^, the higher sorption is may be related to the cation-exchange between Fe^3+^ and H^+^ of –OH and –COOH of CCS or the electrostatic attraction between Fe^3+^ and (-Ve) charge of cellulosic material or phosphoric acid.

In addition to, the preferential sorption of Fe^3+^ over Mn^2+^ at pH 2.6 can be explained by; (a) the ionic radius of the metals has a vital impact on the adsorption mechanism. Where, the ionic radius of Fe^3+^ (0.645Å) is lower than of Mn^2+^ (0.83Å). this leads to the smaller ionic radius of Fe^3+^ can fill the pores of CCS adsorbent and diffuse within it more easily than Mn^2+^^49^. (b) Because of the microporous nature of utilized CCS adsorbent, the tiny Fe^3+^ ions are more easily trapped into the pores of the CCS material^50^.

#### Shaking time study

**Fig. 5:**
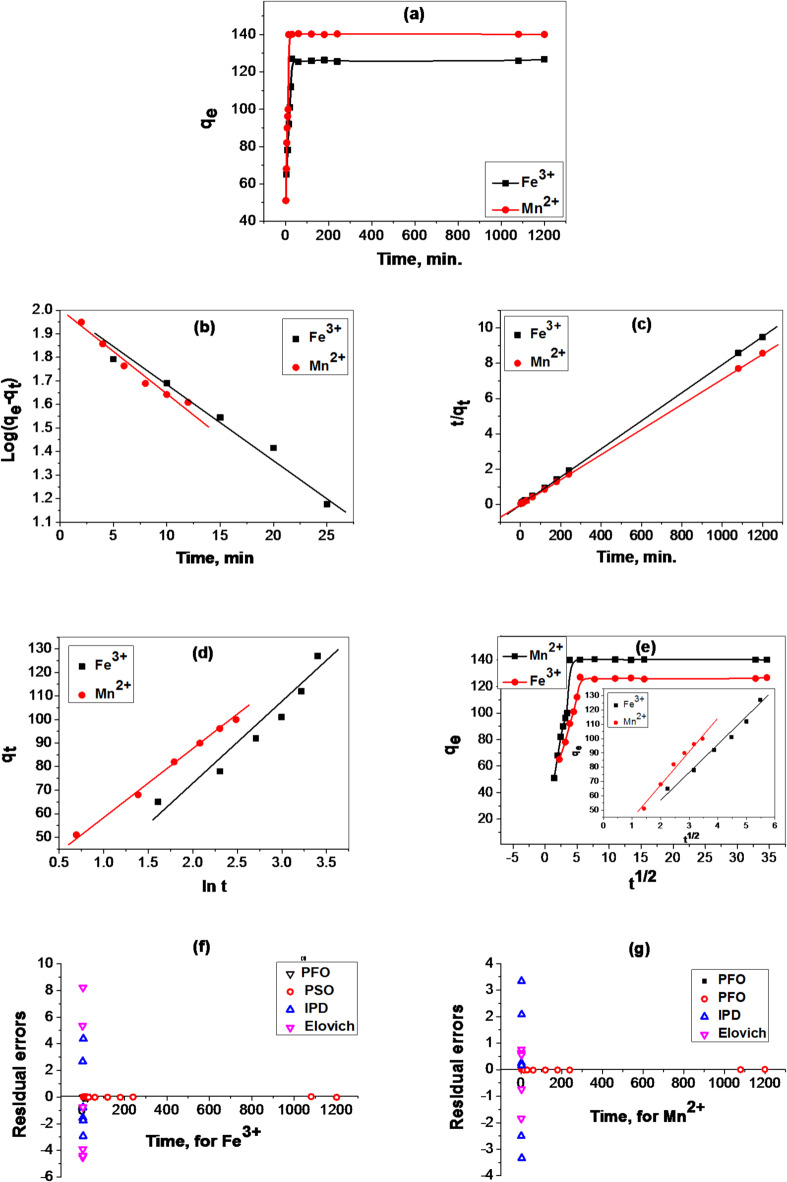
(**a**) Equilibration curves at (C_i_ = 30 mg/L, m = 10 mg, V = 50 ml, T = 25 °C, pH = 2.6 for Fe^3+^ and 5.5 for Mn^2+^), (**b**) Pseudo-first-order, (**c**) Pseudo-second-order, (**d**) Elovich, (**e**) Intra- particle diffusion models, and (**f**,**g**) Residual errors for adsorption of Fe^3+^ and Mn^2+^, respectively onto CCS.

The relationship between shaking time and the adsorption of Fe^3+^ and Mn^2+^-ions on CCS was investigated as seen in Fig. 5a. 50 ml of metal ion solution (30 mg/L) was shaken with 10 mg of adsorbent at various time periods between two minutes and twenty hours. Figure 5a shows the variation of adsorption capacity (q_e_) with shaking time. Maximum adsorption was obtained from the initial 15 min. for Fe^3+^ and 30 min. for Mn^2+^ and steady state was obtained until 20 h. shaking time. This was due to the metal ions (Fe^3+^ and Mn^2+^) occupied the available active sites on the surface of CCS adsorbent^35^. i.e. equilibrium state was obtained from the initial shaking time 30 min. for both Fe^3+^ and Mn^2+^.

### Kinetic models

The values of kinetic parameters of linear regression fitting plots of PFO, PSO, IPD, and Elovich models; Fig. 5b–e, in addition to error functions data are depicted in Table 4. This table indicated that the computed q_e_ values from PSO for Mn^2+^ and Fe^3+^ (140.449 and 126.742 mg/g, respectively**)** are very closer to the experimental values (140 and 127 mg/g, respectively**)** comparing to the other models. Also, the highest R^2^ and the lowest values for RSS, EMS and SD were for pseudo-second order for the two metal ions (Mn^2+^ and Fe^3+^), pointing to the expected sorption process was depended on chemisorptions mechanism. As a result, the pseudo-second order model is progressed the best fit model for sorption of Mn^2+^ and Fe^3+^ onto CCS. Furthermore, Fig. 5d of Elovich model is illustrated that the graph donated rectum line with rise correlation coefficient values for Mn^2+^ and Fe^3+^-ions (0.99838 and 0.96968, respectively), indicating that the Elovich equation can be utilized to describe the kinetics of Mn^2+^ and Fe^3+^ sorption onto CCS, especially for Mn^2+^-ions. It showed good agreement and compliance with the pseudo-second-order model and indicated the chemisorption kinetic model^51^. As depicted in Fig. 5e, the straight lines from plotting qt against t^1/2^ did not crossing the origin point, so, the intraparticle diffusion model was not the rate-determining step in the sorption process of Mn^2+^ and Fe^3+^- ions on CCS and thus, the sorption mechanism might be a combination of film diffusion and intra-particle diffusion indicating the rate-determining step^35^.

To evaluate the fitting applicability of the isotherm models, the residual errors should be applied. Figure 5f,g denoted that the PSO model have lower residual error than the other used models for the sorption of Fe^3+^ and Mn^2+^ onto CCS adsorbent, respectively.

**Table 4:** Kinetic models and error function data for the sorption of Fe^3+^ and Mn^2+^ onto CCS.

Metal ions	Pseudo-first order						
	q_exp_ (mg/g)	k_1_ (min.^−1^)	q_e_ (mg/g)	*R* ^2^	Residual sum of squares (RSS)	Error mean square (EMS)	Standard deviation (SD)
Mn^2+^	140	0.0797	98.6529	0.9593	0.00283	7.06569 × 10^− 4^	0.02658
Fe^3+^	127	0.0694	94.595	0.96779	0.00563	0.00188	0.04331

Metal ions	Pseudo-second order						
	q_exp_ (mg/g)	k_2_ (g mg^−1^min^− 1^)	q_e_	*R* ^2^	Residual sum of squares (RSS)	Error mean square (EMS)	Standard deviation (SD)
Mn^2+^	***140***	0.003	***140.449***	***0.99997***	***0.00275***	***2.29141 × 10*** ^***− 4***^	***0.01514***
Fe^3+^	***127***	0.0026	***126.742***	***0.99996***	***0.00439***	***4.39171 × 10*** ^***− 4***^	***0.02096***

Metal ions	Elovich						
	β (g mg^− 1^)		$\:{\alpha\:}$ (mg g^− 1^ min^− 1^)	*R* ^2^	Residual sum of squares(RSS)	Error mean square (EMS)	Standard deviation (SD)
Mn^2+^	0.0356		84.34	0.99595	5.63324	1.40830	1.18671
Fe^3+^	0.0303		40.23	0.96968	151.85669	37.96417	6.16151

Metal ions	Intra-particle diffusion						
	k_id_ (mg g^−1^min^− 0.5^)		C	*R* ^2^	Residual sum of squares (RSS)	Error mean square (EMS)	Standard deviation (SD)
Mn^2+^	24.29706		19.14892	0.97631	32.96487	8.24122	2.87075
Fe^3+^	18.60734		20.72063	0.97986	40.97545	10.24386	3.2006

The highest R^2^ and the lowest RSS, EMS, and SD are in bold and italic.

#### Influence of temperature and evaluation of thermodynamic parameters

**Fig. 6:**
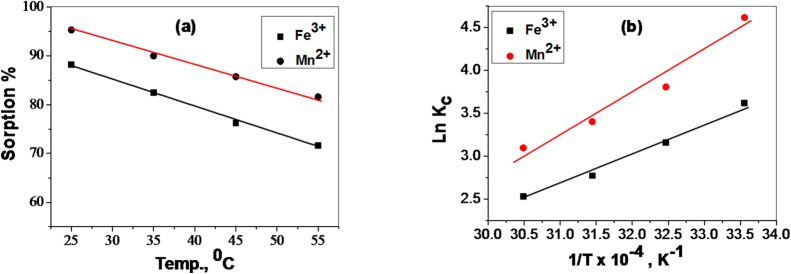
(**a**) Effect of temperature (C_i_ = 30 mg/L, m = 10 mg, V = 50 ml, t = 30 min., pH = 2.6 for Fe^3+^ and 5.5 for Mn^2+^) and (**b**) Van’t Hoff plot for adsorption of Fe^3+^ and Mn^2+^ on CCS.

The influence of various temperatures (25–55 °C) on the interaction between both Fe^3+^ and Mn^2+^-ions and CCS sorbent was clarified in Fig. 6a. It was revealed that when the temperature rises, the adsorption of the metal ions under investigation diminishes from 95.28 to 81.56% for Mn^2+^, and from 88.2 to 71.59% for Fe^3+^, indicating the exothermic nature of the sorption process^52,53^. To determine the thermodynamic behavior for the sorption of Fe^3+^ and Mn^2+^ onto CCS, different thermodynamic parameters, i.e., Gibbs free energy (ΔG^o^), enthalpy (ΔH^o^), and entropy (ΔS^o^), were calculated from Eqs. (3–6) and tabulated in Table 5,

3 $$\:\varDelta\:\mathrm{G}^\circ\:\:=-\:\mathrm{R}\mathrm{T}\:\mathrm{L}\mathrm{n}\:{\mathrm{K}}_{\mathrm{C}}\:,\:{\mathrm{K}}_{\mathrm{C}}\:=\:\frac{{\mathrm{q}}_{\mathrm{e}}}{{\mathrm{C}}_{\mathrm{e}}}$$

4 $$\:\varDelta\:\mathrm{G}^\circ\:\:=\varDelta\:\mathrm{H}^\circ\:-\:\mathrm{T}\varDelta\:\mathrm{S}^\circ\:$$

5 $$\:-\:\mathrm{R}\mathrm{T}\:\mathrm{L}\mathrm{n}\:{\mathrm{K}}_{\mathrm{C}}\:=\varDelta\:\mathrm{H}^\circ\:-\:\mathrm{T}\varDelta\:\mathrm{S}^\circ\:$$

6 $$\:{\mathrm{L}\mathrm{n}\:\mathrm{K}}_{\mathrm{C}}=\frac{\varDelta\:\mathrm{S}^\circ\:}{\mathrm{R}}\:-\:\:\frac{\varDelta\:\mathrm{H}^\circ\:}{\mathrm{R}}\left(\frac{1}{\mathrm{T}}\right)\:$$

where, K_c_ is the equilibrium constant, T is the absolute temperature in Kelvin and R is the universal gas constant (8.314 J.mol^− 1^.K^− 1^). The values of ΔH° and ΔS° were determined from plotting the relationship between Ln Kc and 1/T (Fig. 6b), where the slope of the line refers to (−ΔH°/R), while the intercept represents (ΔS°/R). The values of thermodynamic parameters for the sorption of Fe^3+^ and Mn^2+^ on CCS are illustrated in Table 5. According to this table, the adsorption reaction is exothermic depending on the negative enthalpy (ΔH^o^) values of both Fe^3+^ and Mn^2+^-ions. The changes in free energy (ΔG°) for Fe^3+^ and Mn^2+^ sorption on CCS were exhibit negative values, indicating that the sorption of metal ions onto CCS is feasible and spontaneous^54,55^. Furthermore, the sorption process of the studied metal-ions on CCS becomes physical sorption if the value of ΔG° is lower than − 20 Kj mol^− 1^. While, a ΔG^o^ value higher than − 20 Kj.mol^− 1^, indicates chemisorption. In our findings, the ΔG^o^ values were − 8.971, − 8.085, − 7.330, and − 6.899 Kj. mol^− 1^ for Fe^3+^ and − 11.432, − 9.745, − 8.991, and − 8.443 Kj mol^− 1^ for Mn^2+^, revealing that the sorption nature of Fe^3+^ and Mn^2+^-ions onto the CCS sorbent is chemisorption^54,55^. On the other side, the (+ Ve) value of entropy (ΔS°) refers to an excess randomness pattern on the liquid/solid interface during the sorption process, and this suggests a greater affinity of the CCS adsorbent towards the metal ions. From Table 5, it was noticed that ΔS°-value of Mn^2+^ (100.34 j mol^− 1^ K^− 1^) > Fe^3+^ (70.28 j mol^− 1^ K^− 1^), indicating that the CCS adsorbent has an affinity for Mn^2+^ > Fe^3+^^54^.

**Table 5:** Thermodynamic parameters for the sorption of Fe^3+^ and Mn^2+^-ions on CCS at different temperatures.

Temp., K	ΔG°, kj mol^− 1^		ΔH°, kj mol^− 1^		ΔS°, j mol^− 1^ K^− 1^	
	Fe^3+^	Mn^2+^	Fe^3+^	Mn^2+^	Fe^3+^	Mn^2+^
298	− 8.971	− 11.432			70.281	100.347
308	− 8.085	− 9.745	− 29.819	− 40.543		
318	− 7.330	− 8.991				
328	− 6.899	− 8.443				

#### Adsorption isotherms

**Fig. 7:**
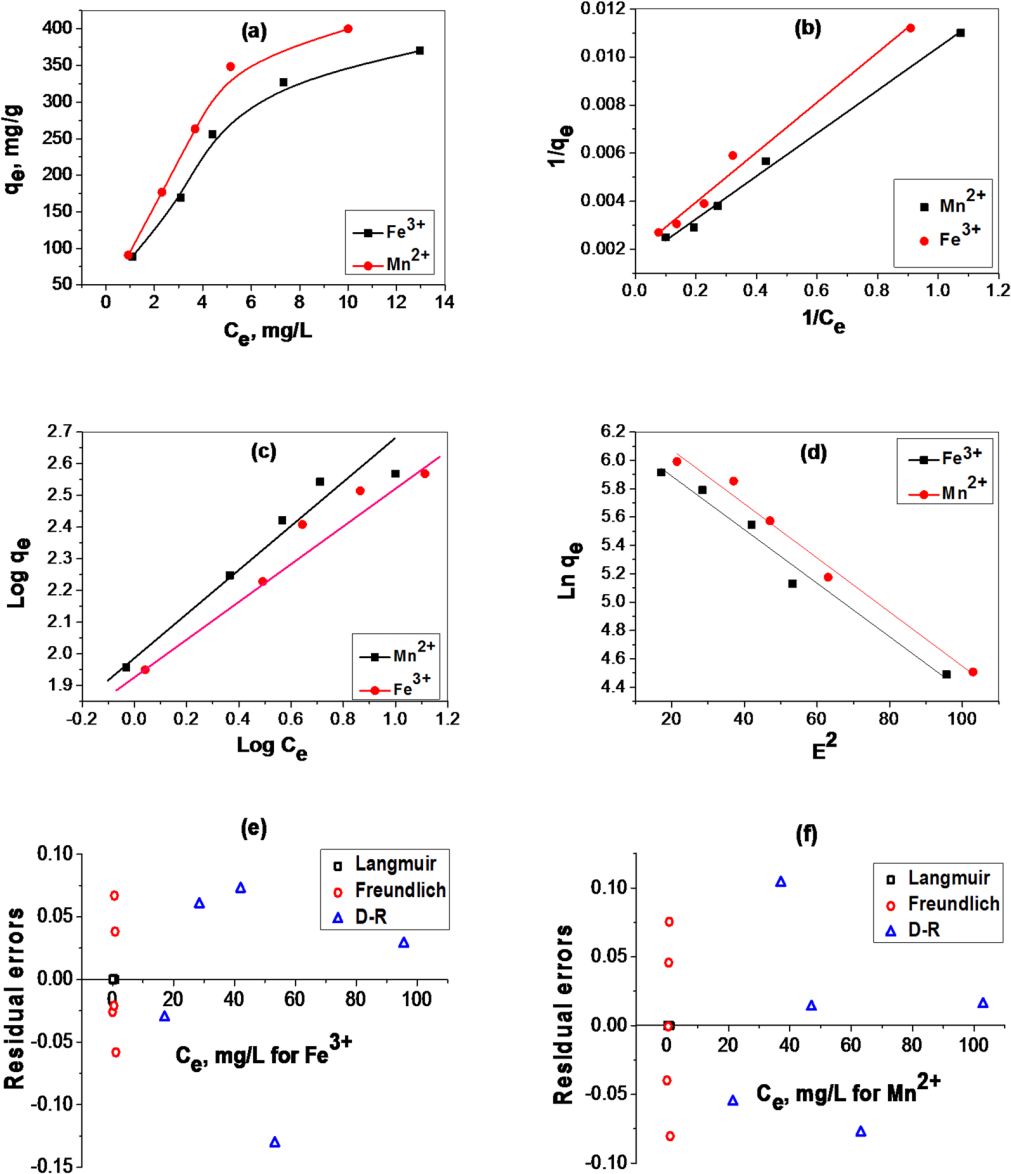
Effect of (**a**) Isotherms (pH = 2.6 for Fe^3+^ and 5.5 for Mn^2+^, m = 10 mg, V = 100 ml, t = 30 min.), (**b**) Langmuir, (**c**) Freundlich, (**d**) D-R, and (**e**,**f**) Residual errors on adsorption of Fe^3+^ and Mn^2+^- ions, respectively onto CCS adsorbent.

The transfer of species from the liquid phase to the surface of the adsorbent particles controls adsorption because diffusion through a solid is naturally slower than a liquid. The process continues until a characteristic equilibrium state is attained when a defined distribution of solute exists between the liquid and solid phases. Under ideal circumstances of 30 min of shaking duration and 10 mg of adsorbent at pH 5.5 and 2.6 for 100 ml Mn^2+^ and Fe^3+^-solution, respectively, the impact of ion concentration on adsorption was investigated. The range of the used ion concentration was 10 mg L^− 1^ to 50 mg L^− 1^. The results are shown in Fig. 7a as plots of q_e_ versus C_e_. It was shown that during the early phases of adsorption, when there were low C_e_ and q_e_ values, all of the isotherms climbed quickly. This behavior suggests that the two adsorbents under study had a large number of active accessible sites. To analyze the adsorption isotherm results, three isothermal models were applied: Langmuir, Freundlich and Dubinin-Radushkevitch (D-R). Figure 7b–d depicts the linear plots of the isothermal models under study, along with their fitting factors and error-functions that utilized to identify the best-fitting data, tabulated in Table 6. It was shown that the values of R^2^ of Mn^2+^ and Fe^3+^ from Langmuir isotherm (Fig. 7b) are 0.9975 and 0.9934, respectively which are higher than R^2^ from Freundlich and D-R models. Furthermore, the Residual sum of squares (RSS), Error mean square (EMS), and Standard deviation (SD) are also included in Table 6. Where, RSS, EMS, and SD from Langmuir are lower than from Freundlich and D-R isotherms for Mn^2+^ and Fe^3+^- ions. This means that the isothermal Langmuir curve is perfectly fit with the experimental results of adsorption of Fe^3+^ and Mn^2+^- ions onto CCS, indicating that the surface is homogeneous and the monolayer capacity of CCS was 680.27 mg/g for Mn^2+^ and 531.9 mg/g for Fe^3+^.

**Table 6:** Langmuir, Freundlich and D-R constants with linear fit correlation coefficient (R^2^) and error function data for the sorption of Fe^3+^ and Mn^2+^ onto CCS.

Metal ions	Langmuir constants						
	q_max_ (mg/g)	b (L/g)	*R* ^2^	Residual sum of squares (RSS)	Error mean square (EMS)	Standard deviation (SD)	
Mn^2+^	680.27	0.1646	***0.9975***	***2.4126 × 10*** ^***− 7***^	***8.04199 × 10*** ^***− 8***^	***2.83584 × 10*** ^***− 4***^	
Fe^3+^	531.9	0.1811	***0.9934***	***6.3964 × 10*** ^***− 7***^	***2.1321 × 10*** ^***− 7***^	***5.04697 × 10*** ^***− 4***^	

Metal ions	Freundlich constants						
	K_f_ (mg/g)	*n*	*R* ^2^	Residual sum of squares (RSS)	Error mean square (EMS)	Standard deviation (SD)	
Mn^2+^	104.08	1.58	0.9682	0.01585	0.00528	0.07269	
Fe^3+^	89.17	1.6	0.9790	0.01044	0.00348	0.059	

Metal ions	D-*R*						
	β (mol^2^ kJ^− 2^)	q_m_ (mg/g)	E, (kJ/mol)	*R* ^2^	Residual sum of squares (RSS)	Error mean square (EMS)	Standard deviation (SD)
Mn^2+^	0.019	637	5.119	0.9810	0.02033	0.00678	0.08231
Fe^3+^	0.0189	526.92	5.143	0.9723	0.02773	0.00924	0.09614

C_i_ (mg/g)	Mn^2+^	Fe^3+^					
	*R*_L_	*R*_L_					
10	0.3779	0.3557					
20	0.2329	0.2164					
30	0.1684	0.1554					
40	0.1318	0.1213					
50	0.1083	0.0994					

The highest R^2^ and the lowest RSS, EMS, and SD are in bold and Italics.

The fundamental property of the Langmuir isotherm was described as a dimensionless-constant separation factor; R_L_, illustrating the isotherm kind and is calculated from Eq. 7^56^. Depending on the R_L_ results (Table 6); it was found that (0 < R_L_<1), indicating that the favorability of the isotherm-reaction^57^.

7 $$\:{\:\:\:\:\:\:\:\:\:\:\:\:\:\:\:\:\:\:\:\:\:\:\:\:\:\:\:\:\:\:\:\:\:\:\:\:\:\:\:\:\:\:\:\:\:\:\:\:\:\:\:\:\:\mathrm{R}}_{\mathrm{L}}=\frac{1}{{(1+\mathrm{b}\mathrm{C}}_{^\circ\:})}\:$$

Based on Table 6, The n-values of both Mn^2+^ and Fe^3+^ are greater than 1, suggesting a favorable and robust adsorption reaction among the CCS surface and the investigated ions^57,58^.

To foretell the reaction mechanism of the studied metal ions with the carbon-cellulosic material, through applying the D-R model (Fig. 7d) and obtaining on the results of mean free energy (E). If E-value is lower than 8 kJ/mol, this indicates a physisorption mechanism, While 8 < E < 16 kJ/mol, refers to ion exchange, but the chemisorption occurs at E > 16 kJ/mol. In this study, E-values of Fe^3+^ and Mn^2+^ are 5.143 and 5.119 kJ/mol, respectively as indicated in Table 6, which are lower than 8 kJ/mol and this is illustrated the physisorption mechanism.

Moreover, the residual errors of the kinetic models fitting in Fig. 7e, f for Fe^3+^ and Mn^2+^ sorption on CCS, respectively, demonstrated that the Langmuir model gives the lowest residual error comparing to the utilized other models.

#### Influence of adsorbent weight

**Fig. 8:**
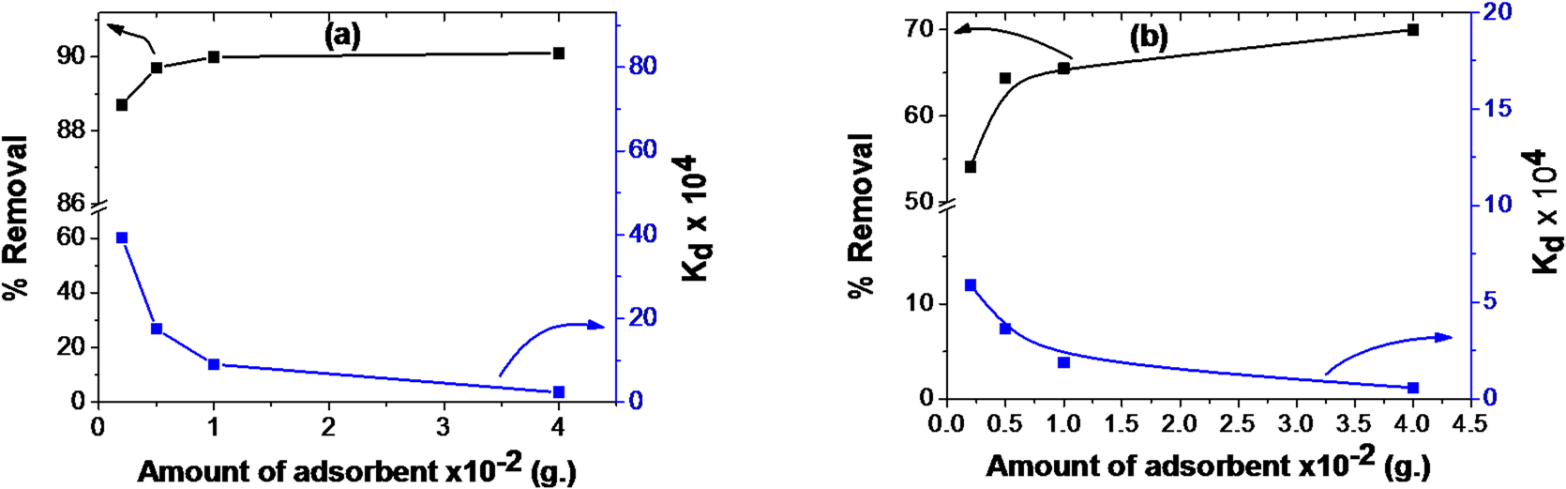
Effect of the amount of adsorbent on the sorption of (**a**) Mn^2+^ and (**b**) Fe^3+^- ions (C_i_= 30 mg/L, V= 50 ml, t = 120 min.).

Adsorption effectiveness is also influenced by the adsorbent weight used, which was tuned by shaking varying amounts from 0.002 to 0.04 g of CCS with 50 ml of either Mn^2+^ or Fe^3+^ at a concentration of 30 mg/L for 120 min., and the outcomes are shown in Fig. 8. The elimination percentages of Mn^2+^ and Fe^3+^ were found to progressively rise from 54.1% to 65.5% and from 88.7% to 90%, respectively, until they reached equilibrium at 0.01 g of CCS. This may be attributed to the presence of many active sites on the adsorbent surface which are filled by the adsorbates. Furthermore, raising the weight of the adsorbent increases the surface area and, thus, the adsorption %^59^. So, just 0.01 g of CCS is needed to remove iron and manganese quantitatively from the utilized aqueous solution. The research experiments were conducted with this quantity of CCS.

## Adsorption mechanism

**Fig. 9:**
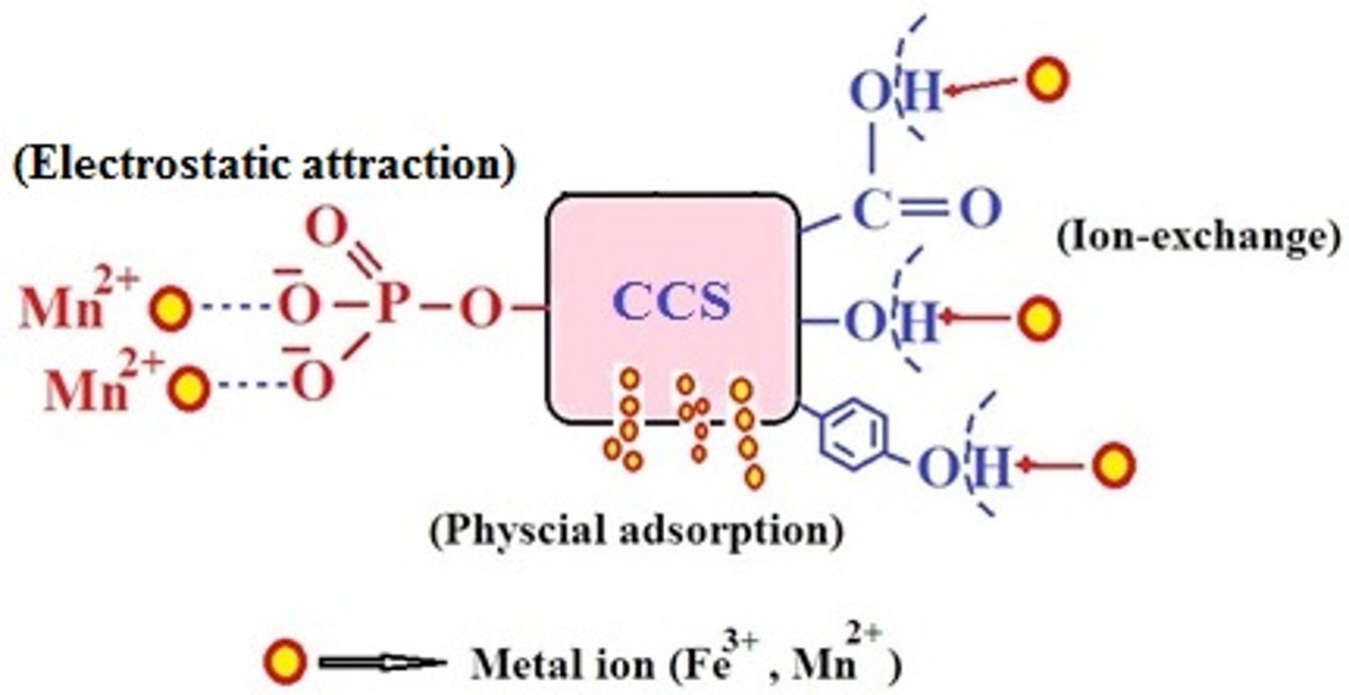
Suggested mechanism of Mn^2+^ and Fe^3+^ on CCS.

**Fig. 10:**
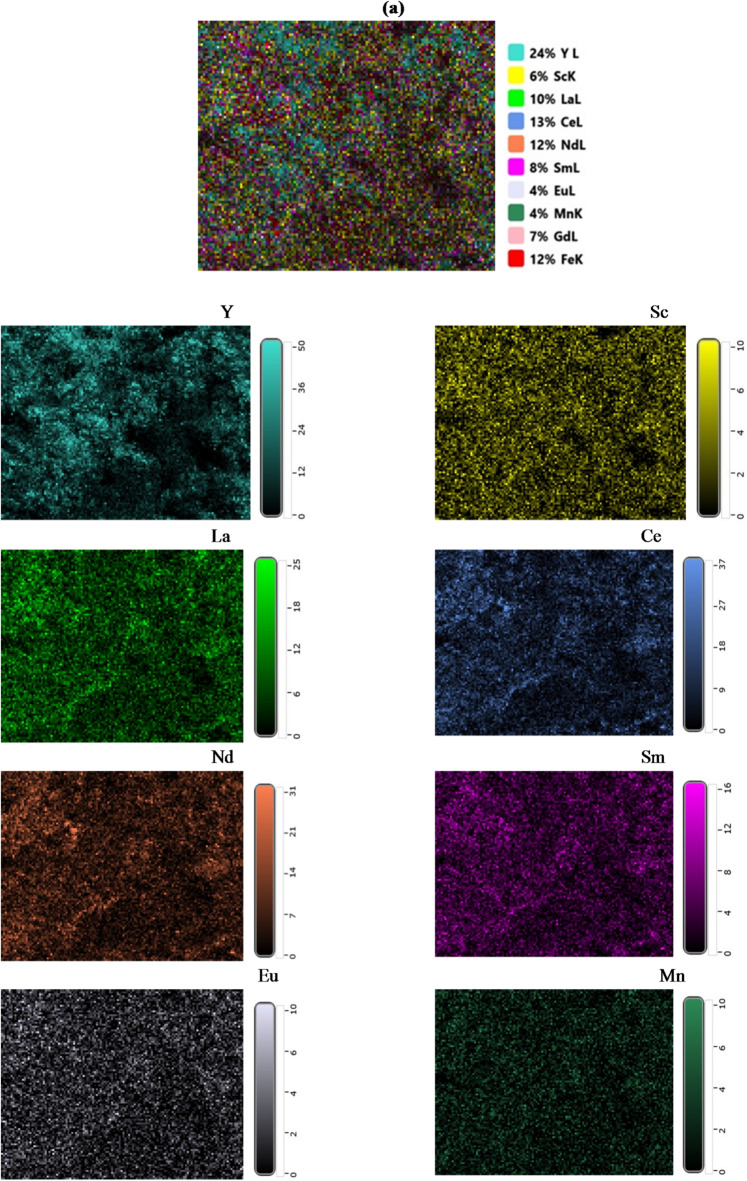

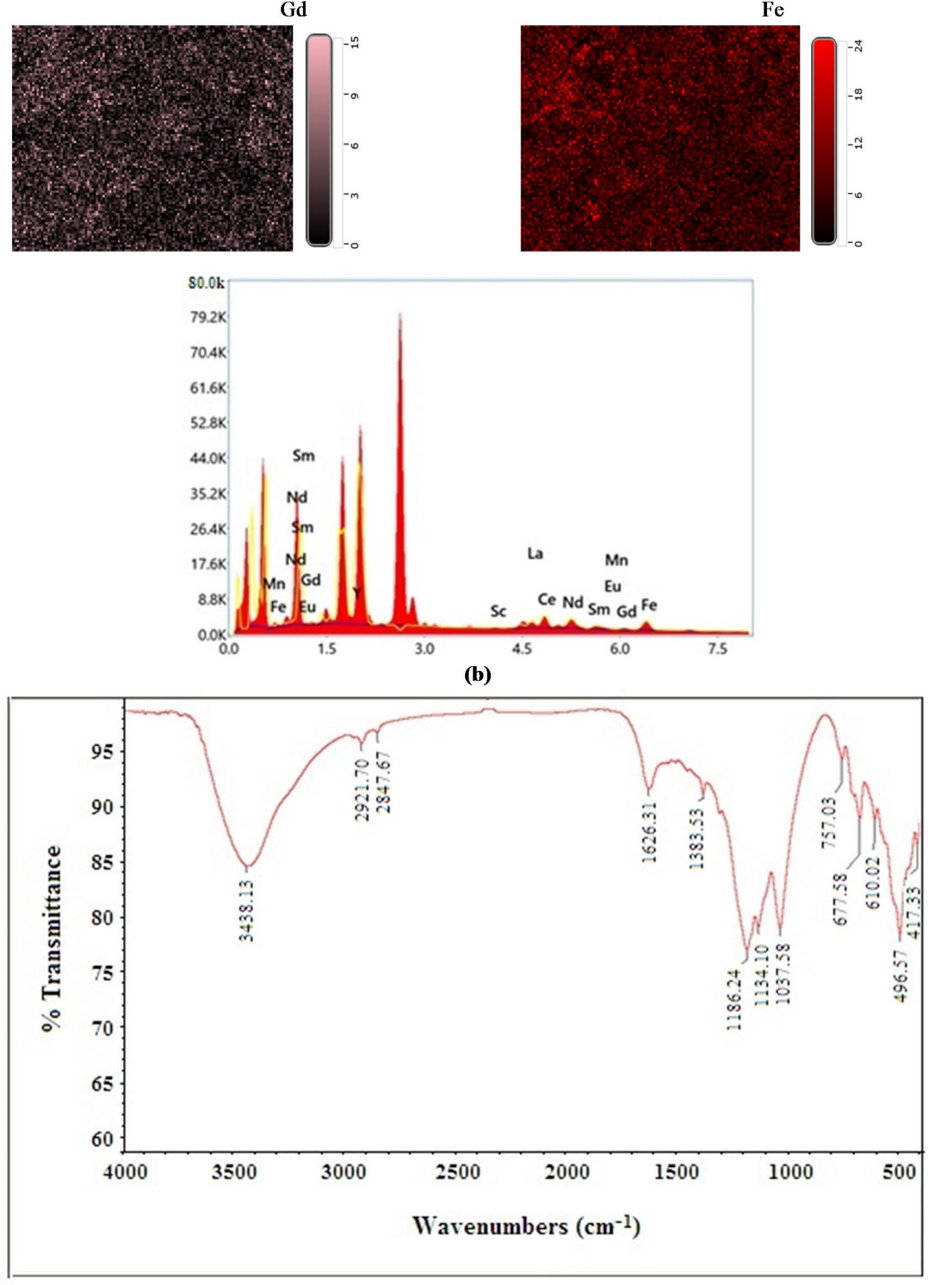
(**a**) EDX-mapping and (**b**) FTIR spectra of loaded CCS with monazite and both Fe^3+^ and Mn^2+^-ions.

The higher adsorption capacity for the CCS is due to different mechanisms as shown in Fig. 9. (1) From FTIR, the higher content of carboxyl, hydroxyl and phenolic groups in the CCS as shown in Fig. 2, leads to interact with the metal ions through ion–exchange mechanism (cation-exchange), forming M-O or P-O-M stretching vibration bond as indicated in Fig. 10b. Furthermore, energy dispersive X-ray (EDX) spectra displayed Fe^3+^ and Mn^2+^-ions, in addition to REEs after their sorption on CCS sorbent (Fig. 10a). (2) Based on the specific surface area (S_BET_) of CCS sorbent, the higher surface area of CCS (831 m^2^/g), increase the pores whereby the studied metal ions were diffused in the pores of the adsorbent surface through physical adsorption. (3) According to the pH study and pHpzc of CCS (3.8), the surface of CCS is (-Ve) charge after pHpzc and the best pH used for sorption of Mn^2+^ is 5.5. So, occur electrostatic interaction between (-Ve) charge of functional groups (as phosphate groups) that exist on the CCS surface and Mn^2+^-ions.

## Actual implementation

The selective sorption and separation of the contaminated Fe^3+^ and Mn^2+^-ions from REEs in monazite leachate liquor was evaluated using a CCS biosorbent through agitating 10 ml of the monazite liquor with 0.2 g of CCS for 30 min at pH 2 and room temperature. Initial; C_i_ and equilibrium; C_e_ concentrations of total REEs, Fe^3+^ and Mn^2+^ were detected employing a Shimadzu, UV-160 A, UV–visible spectrophotometer, Japan. The results of sorption efficiency (%), distribution coefficient (K_d_) and separation factors (SF) were calculated from Eqs. (1, 8, and 9), respectively, and illustrated in Table 7. This table shows that there is almost little sorption of REEs onto CCS. Conversely, Fe^3+^​displayed high sorption ability, but Mn^2+^ clarified noticeably less sorption. This conduct is explained by the differences in ionic radii, where Fe^3+^ (0.645 Å) < Mn^2+^ (0.83 Å) < REEs (1.032–0.861 Å), allowing Fe^3+^ to penetrate more deeply into the pores of the adsorbent surface compared to the larger metal ions. Moreover, the separation factor of Fe^3+^/REEs was significantly higher than that of Mn^2+/^REEs, which may be due to the high concentration of Fe^3+^- ions in monazite leachate.

The sorption performance was confirmed by two tools: (a) energy dispersive X-ray (EDX) spectra as depicted in Fig. 10a, which demonstrated the presence of REEs, Fe^3+^ and Mn^2+^ on the CCS surface. (b) FTIR spectroscopy of the monazite-loaded CCS sorbent (Fig. 10b) demonstrated that its distinctive peaks are almost identical to those of the original sorbent (Fig. 2), with appearance of (1) some minor shifts; specifically, peaks shifted from 507.66 cm^− 1^ to 496.57 cm^− 1^, 735.35 cm^− 1^ to 757 cm^− 1^, and 1062.58 cm^− 1^ to 1037.56 cm^− 1^, and (2) new peak at 610 cm^− 1^, evidence on the sorption of the studied metal ions and REEs from leach fluid through M-O and P-O-M bonds formation via ion exchange reaction. These findings highlight the potential of the CCS sorbent as a highly effective bio-material for the high selectivity separation of Fe^3+^ from REEs of monazite leachate liquor compared to the separation of Mn^2+^ from REEs.

8 $$\:{\mathrm{K}}_{\mathrm{d}}=\:\frac{{\mathrm{q}}_{\mathrm{e}}}{{\mathrm{C}}_{\mathrm{e}}}\:\:\:\:$$

9 $$\:{\mathrm{S}\mathrm{F}}_{\left(\frac{\mathrm{F}\mathrm{e}\:\mathrm{o}\mathrm{r}\:\mathrm{M}\mathrm{n}}{\mathrm{R}\mathrm{E}\mathrm{E}\mathrm{s}}\right)}=\frac{{\mathrm{K}}_{\mathrm{d}\:\left(\mathrm{F}\mathrm{e}\:\mathrm{o}\mathrm{r}\:\mathrm{M}\mathrm{n}\right)}\:}{{\mathrm{K}}_{\mathrm{d}\:\left(\mathrm{R}\mathrm{E}\mathrm{E}\mathrm{s}\right)}}\:$$

**Table 7:** Sorption % and separation factor for extraction Mn^2+^ and Fe^3+^ from REEs of monazite ore.

	Total rare earth elements (REEs)	Fe^3+^	Mn^2+^
Initial concentration (Ci) (mg/g)	11175.89	1480	17.45
Sorption %	0.4	91.5789	1.0294
Distribution coefficient (K_d_)	0.2	45.7894	0.5147
Separation factor (SF)$\:\frac{{\mathrm{F}}_{\mathrm{e}}}{{\mathrm{R}\mathrm{E}\mathrm{E}}_{\mathrm{s}}}$	–	**228.947**	–
Separation factor (SF)$\:\frac{{\mathrm{M}}_{\mathrm{n}}}{{\mathrm{R}\mathrm{E}\mathrm{E}}_{\mathrm{s}}}$	–	–	2.5735
Separation factor (SF)$\:\frac{{\mathrm{F}}_{\mathrm{e}}}{{\:\mathrm{M}}_{\mathrm{n}}}$	–	88.96	–

The highest SF-value is in bold.

## Comparison with other adsorbents

Table 8 compares the manufactured CCS material with other sorbents published in the scientific literature regarding the adsorption of Fe^3+^ and Mn^2+^ ions from aqueous media and rare earth elements (REEs) in monazite leachate liquor. Table 8a showed that the studied CCS material has a higher adsorption capacity for Fe^3+^ and Mn^2+^, as individual metal ions, compared to the other published sorbents. This may be due to the high surface area of CCS material. While, Table 8b, from the monazite liquid, clarified that the CCS material used in this study exhibits high removal efficiency for Fe^3+^ and low removal efficiency for total REEs compared to the adsorption of Fe^3+^ on the (Ca/Alg–GO) nanocomposite and in the SA-g-(PAA/CuO) nanocomposite, Fe^3+^ precipitate, while higher adsorption efficiency for the metal ions of REEs are observed on both the (Ca/Alg–GO) and SA-g-(PAA/CuO) nanocomposites. This indicates a relatively high separation factor for Fe^3+^/REEs for the CCS material used in this research.

**Table 8:** (**a**) AC-based adsorbents for removal of Fe^3+^ and Mn^2+^ and other heavy metal-ions pollutants, and (**b**) Adsorbents utilized for purification of REEs from Fe^3+^-ions.

(a) Adsorbents	Pollutant	q_max_ (mg/g)	pH	Temp. (°C)	Time	*R*%	Year	Reference
CCS	Fe^3+^	531.9	2.6	25	30 min.	85		This work
	Mn^2+^	680.27	5.5			97		
Cellulose adsorbent produced from brewer’s spent grain	Mn^2+^	52.9	6.5	–	15 min.	83.9	2022	^60^
	Pb^2+^	272.5	6.5	–		87.4		
Tannic acid–tethered cellulose	Mn^2+^	32.2	9	–	30 min.	99	2024	^61^
Orange-AC	Mn^2+^	10.604	6	25	120 min.	92.13	2025	^62^
Langsat-AC		11.429				90		
Lemon-AC		10.989				89		
Banana peel biochar modified with nitrilotriacetic acid	Cu^2+^	176.47	6.5	–	–	100	2026	^63^
	Co^2+^	57.58				93.78		
	Mn^2+^	225.19				72.65		
AC(P-2)	Fe^3+^	72.2	3	25	120 min.	90.12 83.42	2021	^64^
	Mn^2+^	49.6	6	25				
Activated carbon prepared from Bombax ceiba fruit shell	Fe^3+^	37.16	3.5	25	25 min.	99.10	2023	^22^
Activated carbon produced from rice husk	Fe^3+^	294.12	3	25	30 min.	94	2013	^65^
	Cu^2+^	666.66				62		
	Pb^2+^	208.33				83		
	Zn^2+^	714.28				22		
Periwinkle shell activated carbon	Fe^3+^	48.251	3	30	20 min.	–	2018	^66^

(b) Adsorbents of REEs	Pollutant	q_max_ (mg/g)	pH	Separation factor (SF)	Temp. and Time (min.)	*R*% (from monazite)	Year	Reference
CCS	Total REEs	–	2	–	25**°**C, 30	0.4		This work
	Fe^3+^	–		SF_Fe/REEs_= 228.9		91.57		
	Mn^2+^	–		SF_Mn/REEs_= 2.6		1.02		
PAn/Tafla	Fe^3+^	190	2	–	25**°**C, 360	–	2019	^67^
	U^6+^	3.65		SF_Fe/U_ = 80.3		–		
	Th^4+^	3.65		SF_Fe/Th_ = 306.3		–		
	Ce^3+^	3.65		SF_Fe/Ce_ = 4900		–		
	La^3+^	4.10		SF_Fe/La_ = 4900		–		
(Ca/Alg–GO) nanocomposite		For Single				*1st cycle*	2021	^26^
	Fe(III)	139	2	No separation for REEs	25**°**C, 240	30		
	U(VI)	129				27		
	Th(IV)	418				65		
SA-g-(PAA/CuO) nanocomposite		For Single						
	La^3+^	83.06	4.5	SF_La/Y_ = 40.66	25**°**C, 5 h.	34.78	2022	^68^
	Ce^3+^	91.49		SF_La/Fe_ = 3.78		58.66		
	Eu^3+^	97.66		SF_Ce/Y_ = 43.32		> 99		
	Y^3+^	7.52		SF_Ce/Fe_ = 4.03		15.22		
	Fe^3+^	24.66		SF_Eu/Y_ = 50.44		Precipitate		
				SF_Eu/Fe_ = 4.69				

## Conclusion

In this article, CCS was synthesized by chemical activation with phosphoric acid and carbonization at 420 °C with gradually rate of 50 °C/10 min. and hold time 1 h. Under these circumstances, CCS displayed high surface area of 831 m^2^.g^− 1^ with micropores percentage (93.27%) and FTIR analysis illustrated the presence of different oxygenated functional groups and phosphate groups such as O–H, C = O, COOH, P = O, P–O–P, P–O–C on the adsorbent surface that rise its sorption efficiency. Where, CCS exhibited 97% and 90.5% of Mn^2+^ and Fe^3+^ ions at pH 5.5 and 2.6 for 30 min., respectively. The kinetic and isotherm results fitted the PSO and Langmuir models, revealing to chemisorption reaction with excellent monolayer sorption capacities of 680.27 and 531.9 mg g^− 1^ for Mn^2+^ and Fe^3+^, respectively, which were more than the adsorption capacities found in the literature. Thermodynamic parameters clarified that the sorption reaction is exothermic and spontaneous.

REEs are vastly utilized in many advanced industries, while their efficiencies are often compromised by the presence of trace heavy metal ions. Therefore, CCS was employed to purify the REEs from the heavy metal ions (Mn^2+^ and particularly, Fe^3+^), as Fe^3+^ is the main contaminating element in REEs fluid. The objective was to achieve an aqueous solution with high REEs concentration and low Fe concentration to obtain on the maximum separation and purification process. CCS exhibit high selectivity and sorption efficiency of Fe^3+^ from REEs of monazite liquor, achieving 91.58% under optimal conditions at pH 2 for 30 min. and at room temperature, while, reached to 0.4% for total REEs. The highest SF of Fe/REEs reached to 228.9, while for Mn/REEs was 2.6, due to the low pH used. Based on the values of ΔS^o^, ΔG^o^, and FTIR analysis, the suggested mechanisms are chemisorption and electrostatic attraction. These results highlight the transformation of CS waste into CCS as an efficient, cost-effective, and highly functional adsorbent used in wastewater treatment, REEs purification from monazite leachate liquor, and protection the environment.

## Data Availability

The datasets used and/or analysed during the current study available from the corresponding author on reasonable request.
